# Mechanics in the Production of Mandibular Fractures: A Clinical, Retrospective Case-Control Study

**DOI:** 10.1371/journal.pone.0149553

**Published:** 2016-02-22

**Authors:** Haihua Zhou, Kun Lv, Rongtao Yang, Zhi Li, Zubing Li

**Affiliations:** 1 The State Key Laboratory Breeding Base of Basic Science of Stomatology (Hubei-MOST) & Key Laboratory of Oral Biomedicine Ministry of Education, School & Hospital of Stomatology, Wuhan University, Wuhan, Hubei, People’s Republic of China; 2 Department of Oral and Maxillofacial Surgery, College and Hospital of Stomatology, Wuhan University, Wuhan, Hubei, People’s Republic of China; Medical University of South Carolina, UNITED STATES

## Abstract

As the mandible is susceptible to fracture, the aim of this study was to use multivariate logistic regression analysis to identify and distinguish various internal factors that may influence the location of mandibular fractures. The study included 1131 patients with maxillofacial fractures during the period from January 2000 to December 2009 to evaluate the association of mandibular fracture location (unilateral symphysis, body, angle, condylar, or bilateral condylar fractures) with various internal factors. Among the 1131 patients, 869 had mandibular fractures. Data on age, sex, soft tissue injuries, dental trauma, and maxillofacial fracture type were collected and analyzed using multivariate logistic regression. In total, 387, 210, 139, 319, and 172 patients were diagnosed with unilateral symphysis, body, angle, unilateral, or bilateral condylar fractures, respectively. The dental trauma in patients with bilateral condylar fractures differed from that in patients with unilateral condylar fractures. Patients with mandibular fracture (unilateral symphysis, body, unilateral or bilateral condylar) possessed an approximately equal risk of soft tissue injuries in the mandible. Patients with either unilateral or bilateral condylar fractures were associated with a low risk of mandibular angle fracture (OR < 1). Similarly, patients with mandibular angle fracture were associated with a low risk of unilateral or bilateral condylar fractures (OR < 1). Moreover, patients with symphysis fracture were associated with a low risk of bilateral condylar fractures (90 of 387 [23.3%], OR 0.899). By contrast, patients with bilateral condylar fractures were associated with a high risk of symphysis fracture (90 of 172 [52.3%], OR 17.38). Patients with condylar fractures, particularly those with bilateral condylar fractures, were infrequently associated with secondary mandibular fractures. Mandibular fractures tended to have less of an association with midfacial fractures. The occurrence of mandibular fractures is strongly correlated with age, sex, soft tissue injuries, dental trauma, and the pattern and position of the maxillofacial fractures in patients.

## Introduction

As the only mobile bone of the facial skeleton, the mandible is vulnerable to fracture because of its mechanically weak components, including the angle, the condylar process, and both sides of the mentum [1–3]. With a fracture incidence rate of 23.8% to 81.3% [4–6], mandibular fractures are the most common of all maxillofacial fractures. Elements which reportedly influence the location of mandible fractures include external factors such as site of impact, direction, and severity of the force of impact [7,8]; internal factors include mouth opening [9–11], dental states such as third molar impaction [3,10,12–17], and intrinsic bone attributes such as physiological atrophy, osteoporosis, and pathological processes [18].

A few studies explored the mechanism by which mandibular fractures occur [7,9,10,19], however, external factors such as the magnitude and direction of the impact, and the shape of the object delivering the impact are widely variable. In addition, these studies cannot control for internal factors, which include condition of the dentition, the position of the mandible, and the influence of associated soft tissue. Thus, only clinical impressions and opinions have served as the basis by which to elucidate the mechanism underlying fracture occurrence [20]. A comprehensive understanding of the various factors that influence the location of mandibular fractures is important to provide clinical and research data for the effective management of these injuries. This paper is part of an extensive investigation that analyzes the mechanics in the production of mandibular fractures from an internal point of view. Accordingly, this study aims to identify and distinguish these internal factors by using a multivariate logistic regression analysis model.

## Results

Of 1131 patients with maxillofacial fractures, a total of 869 patients sustained mandibular fractures. Many of these patients (491 of 869 [56.5%]) had fracture of the condylar process, followed by 391 patients (45.0%) with symphysis, 222 patients (25.5%) with body, and 143 patients (16.5%) with angle fractures. Patients with bilateral symmetrical mandibular fractures (except for those with bilateral condylar fractures) were excluded from the study, in accordance with the exclusion criteria. Accordingly, 387 patients were diagnosed with unilateral symphysis fracture (for details see S1 Appendix), 210 with unilateral body fracture (for details see S2 Appendix), and 139 with unilateral angle fracture (for details see S4 Appendix). All patients with condylar fractures participated in this study (for details see S3 Appendix and S5 Appendix).

As shown in Table 1, the lower anterior teeth of patients with symphysis fracture were often injured (OR 3.270; 95% CI 2.246–4.762), whereas the associated risk of injury to their lower posterior teeth was only 0.836-fold (95% CI 0.524–1.332). The lower anterior (OR 1.971; 95% CI 1.234–3.149) and lower posterior teeth (OR 1.692; 95% CI 0.992–2.888) of patients who sustained mandibular body fracture were frequently injured (Table 2). The lower anterior (OR 0.471; 95% CI 0.294–0.755) and lower posterior teeth (OR 0.604; 95% CI 0.347–1.053) of patients with unilateral condylar fracture were infrequently injured, whereas the upper posterior teeth sustained a high level of injury (OR 1.697; 95% CI 0.934–3.082) (Table 3). Patients with angle fracture had a low risk of tooth injury in the mandible (lower anterior teeth: OR 0.759; 95% CI 0.386–1.492; lower posterior teeth: OR 0.371; 95% CI 0.142–0.967) (Table 4). Patients who sustained bilateral condylar fractures had a low risk of injury in the lower anterior teeth (OR 0.461; 95% CI 0.264–0.806), whereas the teeth in other quadrants were frequently injured (OR > 1) (Table 5).

**Table 1:** Multivariate logistic regression analysis of patients with unilateral symphysis fractures.

	Patients with unilateral symphysis fracture		
	Present	Absent	
Characteristic	(n = 387)	(n = 740)	OR (95% CI) (adjusted)
Male	315	565	1.577 (1.103–2.255)
Age (years)	30.03±12.93	31.68±13.69	1.006 (0.995–1.017)
Soft tissue injuries in maxilla	145	339	2.061 (1.292–3.289)
Soft tissue injuries in mandible	203	276	1.919 (1.191–3.093)
Soft tissue injuries in maxillofacial	289	562	0.401 (0.224–0.716)
Lower anterior teeth injury	135	104	3.270 (2.246–4.762)
Upper anterior teeth injury	130	136	1.903 (1.306–2.773)
Lower posterior teeth injury	63	94	0.836 (0.524–1.332)
Upper posterior teeth injury	63	99	0.703 (0.442–1.118)
Other body fractures/injury	84	103	2.869 (1.923–4.281)
Bilateral condylar	90	82	0.899 (0.238–3.394)
Unilateral condylar	115	202	0.587 (0.274–1.260)
Body	28	194	0.086 (0.040–0.183)
Angle	49	94	0.618 (0.282–1.353)
Ramus	6	8	2.252 (0.553–9.175)
Coronoid	2	23	0.165 (0.030–0.897)
No mandible (other, n = 0)	115	262	0.647 (0.160–2.606)
Mandible (other, n = 1)	162	290	0.724 (0.314–1.668)
Mandible (other, n≥2)	110	188	NA
Without midfacial (n = 0)	292	382	6.210 (3.736–10.32)
Single-midfacial (n = 1)	29	106	1.107 (0.632–1.938)
Multi-midfacial (n≥2)	66	252	NA

NA = Not applicable.

**Table 2:** Multivariate logistic regression analysis of patients with unilateral mandibular body fractures.

	Patients with unilateral body fracture		
	Present	Absent	
Characteristic	(n = 210)	(n = 909)	OR (95% CI) (adjusted)
Male	165	710	1.363 (0.898–2.070)
Age (years)	30.85±13.53	31.15±13.35	1.004 (0.991–1.016)
Soft tissue injuries in maxilla	67	413	1.049 (0.594–1.853)
Soft tissue injuries in mandible	107	369	1.684 (0.919–3.088)
Soft tissue injuries in maxillofacial	152	694	0.595 (0.293–1.208)
Lower anterior teeth injury	52	183	1.971 (1.234–3.149)
Upper anterior teeth injury	34	227	0.904 (0.554–1.477)
Lower posterior teeth injury	41	112	1.692 (0.992–2.888)
Upper posterior teeth injury	15	142	0.303 (0.152-.604)
Other body fractures/injury	40	142	2.334 (1.458–3.736)
Bilateral condylar	27	143	0.436 (0.097–1.970)
Unilateral condylar	56	260	0.462 (0.191–1.116)
Symphysis	24	363	0.099 (0.044–0.226)
Angle	33	109	0.669 (0.264–1.693)
Ramus	5	9	7.846 (1.718–35.83)
Coronoid	1	24	0.473 (0.051–4.428)
No mandible (other, n = 0)	78	262	1.951 (0.370–10.295)
Mandible (other, n = 1)	92	327	0.945 (0.357–2.505)
Mandible (other, n≥2)	40	320	NA
Without midfacial (n = 0)	165	504	13.620 (6.882–26.96)
Single-midfacial (n = 1)	21	112	2.510 (1.272–4.956)
Multi-midfacial (n≥2)	24	293	NA

NA = Not applicable.

**Table 3:** Multivariate logistic regression analysis of patients with unilateral condylar fractures.

	Patients with unilateral condylar fracture		
	Present	Absent	
Characteristic	(n = 319)	(n = 640)	OR (95% CI) (adjusted)
Male	234	520	0.783 (0.519–1.181)
Age (years)	31.22±15.12	31.83±12.30	0.996 (0.984–1.009)
Soft tissue injuries in maxilla	75	382	0.487 (0.285–0.830)
Soft tissue injuries in mandible	182	185	1.598 (0.893–2.860)
Soft tissue injuries in maxillofacial	225	498	0.748 (0.379–1.477)
Lower anterior teeth injury	58	137	0.471 (0.294–0.755)
Upper anterior teeth injury	81	133	1.651 (1.011–2.694)
Lower posterior teeth injury	41	70	0.604 (0.347–1.053)
Upper posterior teeth injury	55	70	1.697 (0.934–3.082)
Other body fractures/injury	39	120	1.109 (0.676–1.819)
Symphysis	117	184	2.919 (0.929–9.170)
Body	59	134	1.561 (0.500–4.875)
Angle	16	123	0.340 (0.108–1.075)
Ramus	4	8	6.003 (0.950–37.94)
Coronoid	3	21	5.065 (0.806–31.84)
No mandible (other, n = 0)	138	262	20.17 (2.509–162.2)
Mandible (other, n = 1)	158	267	3.722 (1.270–10.91)
Mandible (other, n≥2)	23	111	NA
Without midfacial (n = 0)	271	261	23.21 (12.44–43.30)
Single-midfacial (n = 1)	26	100	2.701 (1.410–5.173)
Multi-midfacial (n≥2)	22	279	NA

NA = Not applicable.

**Table 4:** Multivariate logistic regression analysis of patients with unilateral mandibular angle fractures.

	Patients with unilateral angle fracture		
	Present	Absent	
Characteristic	(n = 139)	(n = 988)	OR (95% CI) (adjusted)
Male	111	768	1.019 (0.543–1.911)
Age (years)	28.18±11.26	31.52±13.69	1.020 (1.000–1.040)
Soft tissue injuries in maxilla	53	429	0.810 (0.350–1.874)
Soft tissue injuries in mandible	55	426	0.362 (0.156-.842)
Soft tissue injuries in maxillofacial	101	752	1.792 (0.661–4.860)
Lower anterior teeth injury	19	220	0.759 (0.386–1.492)
Upper anterior teeth injury	7	258	0.229 (0.082-.634)
Lower posterior teeth injury	13	143	0.371 (0.142-.967)
Upper posterior teeth injury	6	154	1.276 (0.438–3.711)
Other body fractures/injury	24	160	1.228 (0.606–2.489)
Bilateral condylar	3	168	0.402 (0.048–3.351)
Unilateral condylar	15	303	0.449 (0.103–1.957)
Symphysis	47	342	2.952 (0.703–12.39)
Body	32	188	2.430 (0.570–10.37)
Ramus	0	14	NA
Coronoid	3	22	2.525 (0.343–18.59)
No mandible (other, n = 0)	49	0	1.448E18
Mandible (other, n = 1)	79	356	9.084E7
Mandible (other, n≥2)	11	370	1.457E7
Without midfacial (n = 0)	119	555	5.812 (2.065–16.35)
Single-midfacial (n = 1)	6	129	2.026 (0.504–8.145)
Multi-midfacial (n≥2)	14	304	NA

NA = Not applicable.

**Table 5:** Multivariate logistic regression analysis of patients with bilateral condylar fractures.

	Patients with bilateral condylar fractures		
Characteristic	Present	Absent	
	(n = 172)	(n = 640)	OR (95% CI) (adjusted)
Male	127	520	0.734 (0.432–1.246)
Age (years)	28.08±13.85	31.83±12.30	1.025 (1.008–1.043)
Soft tissue injuries in maxilla	27	382	0.216 (0.105-.445)
Soft tissue injuries in mandible	116	185	2.670 (1.199–5.947)
Soft tissue injuries in maxillofacial	132	498	1.001 (0.402–2.488)
Lower anterior teeth injury	46	137	0.461 (0.264–0.806)
Upper anterior teeth injury	53	133	2.108 (1.165–3.813)
Lower posterior teeth injury	47	70	1.835 (0.964–3.496)
Upper posterior teeth injury	37	70	1.452 (0.689–3.061)
Other body fractures/injury	28	120	1.333 (0.721–2.463)
Symphysis	90	184	17.38 (1.718–175.7)
Body	29	134	4.853 (0.487–48.39)
Angle	4	123	0.567 (0.057–5.657)
Ramus	2	8	15.54 (0.688–351.2)
Coronoid	1	21	10.64 (0.464–244.0)
No mandible (other, n = 0)	54	262	458.6 (4.842–4.345E4)
Mandible (other, n = 1)	110	261	23.01 (2.172–243.6)
Mandible (other, n≥2)	8	117	NA
Without midfacial (n = 0)	146	261	7.408 (3.479–15.77)
Single-midfacial (n = 1)	9	100	0.905 (0.363–2.259)
Multi-midfacial (n≥2)	17	279	NA

NA = Not applicable.

The correlations of soft tissue injuries with mandibular fractures are summarized in Tables 1–5. Among the patients who sustained symphysis or mandibular body fractures, soft tissue injury was amplified in both the maxilla and jaw (Tables 1 and 2). However, in patients with mandibular angle fracture, soft tissue injuries were infrequently associated with either the maxilla (OR 0.810; 95% CI 0.350–1.874) or the jaw (OR 0.362; 95% CI 0.156–0.842) (Table 4). For patients with either unilateral or bilateral condylar fractures, soft tissue in the maxilla (unilateral: OR 0.487; 95% CI 0.285–0.830; bilateral: OR 0.216; 95% CI 0.105–0.445) was infrequently injured, whereas soft tissue in the jaw (unilateral: OR 1.598; 95% CI 0.893–2.860; bilateral: OR 2.670; 95% CI 1.199–5.947) sustained a high level of injury (Tables 3 and 5).

Patients with symphysis or mandibular body fracture were at low risk (OR < 1) of other mandibular fractures, except for mandibular ramus fracture, for which they are at high risk (symphysis: OR 2.252; 95% CI 0.553–9.175; body: OR 7.846; 95% CI 1.718–35.83) (Tables 1 and 2). For patients with either unilateral or bilateral condylar fractures, the risk of fracture of other mandibular sites was substantially high (OR > 1), except for mandibular angle fracture (unilateral: OR 0.340; 95% CI 0.108–1.075; bilateral: OR 0.567; 95% CI 0.057–5.657) (Tables 3 and 5). Patients with mandibular angle fracture showed an associated high risk of other mandibular fractures (OR > 1), except for unilateral or bilateral condylar fractures (unilateral: OR 0.449; 95% CI 0.103–1.957; bilateral: OR 0.402; 95% CI 0.048–3.351) (Table 4).

Patients with symphysis fracture showed an associated low risk of unilateral (OR 0.587; 95% CI 0.274–1.260) or bilateral condylar fractures (OR 0.899; 95% CI 0.238–3.394) (Table 1). However, symphysis fracture frequently occurred in patients who sustained condylar fractures, especially those patients with bilateral condylar fractures (unilateral: OR 2.919; 95% CI 0.929–9.170; bilateral: OR 17.38; 95% CI 1.718–175.7) (Tables 3 and 5).

Patients with condylar fractures, especially those with bilateral condylar fractures, showed an infrequent association with secondary mandibular fractures (patients with unilateral condylar fracture: OR 20.17; 95% CI 2.509–162.2; bilateral: OR 458.6; 95% CI 4.842–4.345E4) (Tables 3 and 5). Interestingly, mandibular fractures tended to have less of an association with midfacial fractures (Tables 1–5).

Male patients had greater risk of symphysis fracture than female patients (OR 1.577; 95% CI 1.103–2.255) (Table 1). On average, more younger patients presented with bilateral condylar fractures than older patients (OR 1.025; 95% CI 1.008–1.043) (Table 5).

## Discussion

A thorough understanding of the mechanism and management of mandibular fractures is paramount to oral and maxillofacial surgeons, independent of their level of training. This study analyzed and evaluated the correlation of various internal factors with the location of mandibular fractures. Results showed that the occurrence of mandibular fractures was highly correlated with the age, sex, soft tissue injuries, dental trauma, and pattern and position of the maxillofacial fractures of the patients.

Occlusion and dentition are functions of the mandibular arch and the temporomandibular joint. Dentition and occlusion can partly absorb the external force of a mandibular blow. Previous studies analyzed the association between the state of dentition and the location of mandibular fractures [13,14,19,21]. However, the present study evaluated the relationship of dental injuries to the location of mandibular fracture. Results show that the lower anterior teeth were frequently injured in patients with symphysis fracture. Patients who sustained unilateral condylar fracture also sustained a high degree of injury to the upper posterior teeth. In patients who sustained mandibular body fracture, the lower anterior and lower posterior teeth were frequently injured. By contrast, patients with angle fracture had a low risk of tooth injury in the mandible. Logistic regression analysis was used to control confounding variables, but these findings confirmed our previous conclusion, that teeth near the fracture area are at high risk of being injured [3]. These findings indicate that the dental state in the vicinity of a mandibular fracture should be given paramount importance. However, since the present study was designed retrospectively, the original state of dentition surrounding the fractures is, in many cases, unknown due to the lack dental records. Thus, we could not answer questions pertaining to the following important concerns: 1) whether or not poor dentition leads to increased risk for mandibular fracture, and 2) does poor dentition, combined with a fracture in the area, lead to poor wound healing or increased risks for infection. Further studies are needed to address these issues.

The dental trauma in patients with bilateral condylar fractures reasonably differed from that in patients with unilateral condylar fracture. Previous studies revealed that dental injuries occurred more frequently in patients with bilateral condylar fractures than in those with unilateral condylar fracture [15,22]. Moreover, severe dental injuries are more frequently encountered in bilateral than in unilateral condylar fracture cases [15]. In the present study, the type and location of dental injury significantly differed between patients with bilateral condylar fractures and those with unilateral condylar fracture. The lower posterior teeth of patients with bilateral condylar fractures were more frequently injured than those of patients with unilateral condylar fracture.

Patients with mandibular fractures (symphysis, body, unilateral or bilateral condylar fractures) possessed an approximately equal risk of soft tissue injury in the mandible (OR in ascending order was unilateral condylar, 1.598; body, 1.684; symphysis, 1.919; and bilateral condylar, 2.670). Patients with mandibular angle fracture possessed a significantly low risk of associated soft tissue injury in the mandible (OR 0.362). These findings suggest that the fracture pattern of the mandible (different locations) depends on the effect of an external force and that the magnitude and direction of the force influences the location of mandibular fractures [23]. Directing an external force along the parasymphysis–body region of the mandible usually immediately produces a symphysis or mandibular body fracture. Nonetheless, the mobile condylar process moves away from the impact point until it is limited by the bony fossa and associated soft tissue [20]. Applying a greater external force to the parasymphysis–body region of the mandible produces symphysis–condylar co-fractures (unilateral or bilateral condylar). The present study confirmed that fracture of symphysis was prone to occur in patients with sustained condylar fractures, especially in those with bilateral condylar fractures (unilateral: OR 2.919; bilateral: OR 17.38), but not for those with mandibular angle fracture. Patients with mandibular angle fracture were associated with a low risk of soft tissue injury in the mandible (OR 0.362). The angle of mandible located behind the ramus had minimal possibility of being impacted by an external force. This phenomenon may be attributed to the existence of impacted teeth in the retromolar area of the mandible. The presence of impacted teeth significantly increases the risk of an angle fracture [24–26].

We found an inverse relationship between angle fracture and condylar fractures (unilateral or bilateral). Patients with either unilateral or bilateral condylar fractures had an associated low risk of mandibular angle fracture (OR < 1). Similarly, patients with mandibular angle fracture were associated with a low risk of unilateral or bilateral condylar fractures (OR < 1). We hypothesize that this phenomenon occurs as a consequence of the presence of impacted teeth. Previous studies reported that the presence of impacted teeth in the mandible increases the risk of mandibular angle fracture, while reducing the risk of condylar fractures [27,28].

Patients with symphysis fracture were associated with a low risk of bilateral condylar fractures (90 of 387 [23.3%], OR 0.899). By contrast, patients with bilateral condylar fractures were associated with a high risk of symphysis fracture (90 of 172 [52.3%], OR 17.38). These results are contradictory but interesting. The biomechanics of symphysis fracture is simple; with the application of an external force on the labial side of the symphysis, tensile strain is produced through flattening of the chin and concomitant stretching of the lingual cortical plate [19]. Under these circumstances, fracture of the symphysis occurs more commonly, only a great force applied to the symphysis can pass through the mandibular arch; this phenomenon leads to bilateral condylar fractures. For patients with bilateral condylar fractures, the condylar processes are no longer limited by the glenoid fossa; applying an external force widens the mandibular arch and induces tension along the lingual aspect of symphysis, which is then easily fractured. This event leads to symphysis–bilateral condylar co-fractures. Consequently, in clinical practice, these patients usually present with widening of the mandibular arch, as reflected in the occlusion or the width of the face [29].

A close relationship has been revealed between condylar fractures and other mandibular fractures. However, patients with condylar fractures infrequently demonstrate associated secondary mandibular fractures, especially patients with bilateral condylar fractures. Furthermore, many of the 869 patients with sustained mandibular fractures had fractured condylar processes (491 [56.5%]). The reduced cross-sectional dimension in the sub-condylar predisposed these patients to fracture when a great force was concentrated in these locations [20]. The occurrence of condylar fractures reduced the occurrence of other mandibular fractures. We found a negative correlation suggesting that mandibular fractures tended to have less of an association with midfacial fractures. This is expected because the mandible is located at the lowest position, and, therefore, it is more likely to be struck by an object or hit the ground first (from high or at ground level). Additionally, fracture of the mandible likely serves as a protective mechanism, allowing greater dissipation of forces, resulting in less residual energy to be transmitted to the midfacial bone.

The present retrospective case-control study provided an excellent model to evaluate various internal factors that may influence the location of mandibular fractures. The main advantages of this study are the large size of the study population and the control for confounding variables through multivariate logistic regression. However, the qualitative and intangible nature of many other internal and external factors should be taken into account. At present, little is known about the mechanics in the production of mandibular fractures. These questions have not been answered because of lack of experimental data [19]. As a retrospective clinical study, this work did not record the actual circumstances regarding mouth opening or closing during the occurrence of mandibular fractures. The magnitude and direction of the impact to the mandible were also unknown. These limitations are difficult to avoid. Therefore, a multi-center prospective clinical study or a well-designed experiment on the mechanism of mandibular fracture should be conducted in the future. From the research standpoint, it would be interesting to look at possibly recreating a few simple cases in the lab using synthetic bone (Sawbones or similar products) to determine if certain impacts with a given vectoral direction and magnitude can recreate the same fracture pattern. If this is able to be done, then perhaps the next logical step would be to work backwards (by using CT imaging). Given a certain fracture pattern, one could examine the direction of impact and the magnitude of the force and apply this information to predict the location of other fractures.

In conclusion, the occurrence of mandibular fractures is highly related to the age, sex, soft tissue injuries, dental trauma, and the pattern and position of the maxillofacial fractures of the patients. Through this study, it was expected that these findings could help clinicians to better understand the pathogenesis of mandibular fractures, and to better predict and evaluate the condition of patients with mandibular fractures. It may also help to reduce the incidence of misdiagnosis and missed diagnosis in mandibular fractures. For example, in a patient with poor dentition and bilateral condylar fractures, we must ensure to examine for a symphysis fracture [OR 17.38] and a single fracture in other locations of the mandible [OR 23.01]. Of particular importance is to understand that the mandible fracture does not necessarily have to be gross; a hairline fracture could exist. Finally, this study may also provide clinical and research data for the effective management of these injuries.

## Methods

### Ethics Statement

We conducted a hospital-based retrospective case-control study at Stomatology College and Hospital, Wuhan University, from January 2000 to December 2009. We have read and followed the ethical guidelines of the Declaration of Helsinki in this investigation. All protocol, survey forms, and consent forms were approved by the Institutional Review Board (IRB) of Wuhan University. Written consent provided by the patients was waived by IRB.

### Patient Population and Data Collection

This study included patients with maxillofacial fractures admitted in the Department of Oral and Maxillofacial Surgery, Stomatology College and Hospital, Wuhan University from January 2000 to December 2009. Patients with repeat admissions and incomplete information were excluded from this study. In total, 1131 participants with maxillofacial fractures had complete diagnostic records. Data on age, sex, soft tissue injuries, dental trauma, and maxillofacial fracture type were collected and standardized by an investigator on the basis of the case histories, clinical and radiographic examinations, and medical records of the patients.

Mandibular fractures were classified as condylar (unilateral or bilateral), symphysis, body, angle, ramus, and coronoid fractures.

The site of dental injuries was divided into four groups (upper anterior, lower anterior, upper posterior, and lower posterior teeth). The anterior teeth included incisors and canine, and the posterior teeth included premolars and molars.

Soft tissue injuries in the mandible or maxilla were recorded. Associated fractures such as skull, thoracic, cervical, vertebra, pelvis, extremity, and abdominal injuries, were also recorded as “other body fractures/injuries.”

### Case and Control Groups

Among the 1131 patients, those diagnosed with a certain type of mandibular fractures (unilateral symphysis, body, angle, condylar or bilateral condylar fractures) comprised the case group. Meanwhile, patients with maxillofacial fractures but without a certain type of mandibular fractures comprised the control group.

### Statistical Analysis

Statistical analysis was performed using SPSS version 16.0 (Chicago, IL). The data were analyzed through multivariate logistic regression.

## Supporting Information

S1 Appendix Logistics regression analysis of unilateral symphysis fracture. (XLS)

S2 Appendix Logistics regression analysis of unilateral mandibular body fracture. (XLS)

S3 Appendix Logistics regression analysis of unilateral condylar fracture. (XLS)

S4 Appendix Logistics regression analysis of unilateral angle fracture. (XLS)

S5 Appendix Logistics regression analysis of bilateral condylar fractures. (XLS)

## References

[1] Mitsukawa N, Satoh K, Uemura T, Hosaka Y. (2004) An unusual traumatic fracture of the mandibular symphysis resembling horizontal osteotomy for genioplasty. J Craniofac Surg 15: 229–331. 15167236 10.1097/00001665-200403000-00011

[2] Halazonetis JA (1968) The 'weak' regions of the mandible. Br J Oral Surg;6: 37–48. 5244106 10.1016/s0007-117x(68)80025-3

[3] Zhou HH, Ongodia D, Liu Q, Yang RT, Li ZB. (2013) Dental trauma in patients with single mandibular fractures. Dent Traumatol 29: 291–296. 10.1111/j.1600-9657.2012.01173.x 22882802

[4] Lieger O, Zix J, Kruse A, Iizuka T (2009) Dental injuries in association with facial fractures. J Oral Maxillofac Surg 67: 1680–1684. 10.1016/j.joms.2009.03.052 19615582

[5] Gassner R, Tuli T, Hachl O, Rudisch A, Ulmer H. (2003) Cranio-maxillofacial trauma: A 10 year review of 9,543 cases with 21,067 injuries. J Craniomaxillofac Surg 31: 51–61. 12553928 10.1016/s1010-5182(02)00168-3

[6] Erol B, Tanrikulu R, Gorgun B (2004) Maxillofacial fractures. Analysis of demographic distribution and treatment in 2901 patients (25-year experience). J Craniomaxillofac Surg 32: 308–313. 15458673 10.1016/j.jcms.2004.04.006

[7] Reitzik M, Lownie JF, Cleaton-Jones P, Austin J. (1978) Experimental fractures of monkey mandibles. Int J Oral Surg 7: 100–103. 98455 10.1016/s0300-9785(78)80054-4

[8] Rudderman RH, Mullen RL (1992) Biomechanics of the facial skeleton. Clin Plast Surg 19: 11–29. 1537212

[9] da Fonseca GD (1974) Experimental study on fractures of the mandibular condylar process (mandibular condylar process fractures). Int J Oral Surg 3: 89–101. 4209422 10.1016/s0300-9785(74)80040-2

[10] Petzel JR, Bulles G (1981) Experimental studies of the fracture behaviour of the mandibular condylar process. J Maxillofac Surg 9: 211–215. 6948065 10.1016/s0301-0503(81)80046-x

[11] Cope MR, Lawlor MG (1985) An unusual mandibular dislocation. Br J Oral Maxillofac Surg 23: 112–117. 3158331 10.1016/0266-4356(85)90060-9

[12] Huelke DF, Burdi AR, Eyman CE (1962) Association between mandibular fractures and site of trauma, dentition and age. J Oral Surg Anesth Hosp Dent Serv 20: 478–481. 13955479

[13] Huelke DF (1964) Location of mandibular fractures related to teeth and edentulous regions. J Oral Surg Anesth Hosp Dent Serv 22: 396–405. 14178798

[14] Lindahl L (1977) Condylar fractures of the mandible. I. Classification and relation to age, occlusion, and concomitant injuries of teeth and teeth-supporting structures, and fractures of the mandibular body. Int J Oral Surg 6: 12–21. 402318 10.1016/s0300-9785(77)80067-7

[15] Silvennoinen U, Lindqvist C, Oikarinen K (1993) Dental injuries in association with mandibular condyle fractures. Endod Dent Traumatol 9: 254–259. 7908262 10.1111/j.1600-9657.1993.tb00282.x

[16] Ignatius ET, Oikarinen KS, Silvennoinen U (1992) Frequency and type of dental traumas in mandibular body and condyle fractures. Endod Dent Traumatol 8: 235–40. 1363840 10.1111/j.1600-9657.1992.tb00250.x

[17] Zhou HH, Ongodia D, Liu Q, Yang RT, Li ZB. (2013) Dental trauma in patients with maxillofacial fractures. Dent Traumatol 29: 285–290. 10.1111/j.1600-9657.2012.01169.x 22783913

[18] Krimmel M, Reinert S (2000) Mandibular fracture after third molar removal. J Oral Maxillofac Surg 58: 1110–1112. 11021704 10.1053/joms.2000.9566

[19] Huelke DF (1961) Mechanics in the production of mandibular fractures: A study with the" stresscoat" technique. I. Symphyseal impacts. Journal of dental research 40: 1042–1056. 10.1177/00220345640430031601 14159491

[20] Miloro M, Ghali GE, Larsen PE, Waite P, (2004) Peterson's principles of oral and maxillofacial surgery 2nd edn Hamilton, Ont.; London: BC Decker.

[21] Huelke DF, Alphonse R, Eyman CE. (1961) Mandibular fractures as related to the site of trauma and the state of dentition. Journal of dental research 40:1262–1274.

[22] Zhou HH, Liu Q, Cheng G, Li ZB. (2013) Aetiology, pattern and treatment of mandibular condylar fractures in 549 patients: A 22-year retrospective study. J Craniomaxillofac Surg 41: 34–41. 10.1016/j.jcms.2012.05.007 22727898

[23] Goodday RH (2013) Management of fractures of the mandibular body and symphysis. Oral Maxillofac Surg Clin North Am 25: 601–616. 10.1016/j.coms.2013.07.002 24021623

[24] Fuselier JC, Ellis EE 3rd, Dodson TB (2002) Do mandibular third molars alter the risk of angle fracture? J Oral Maxillofac Surg 60: 514–518. 11988927 10.1053/joms.2002.31847

[25] Subhashraj K (2009) A study on the impact of mandibular third molars on angle fractures. J Oral Maxillofac Surg 67: 968–972. 10.1016/j.joms.2008.09.005 19375005

[26] Zhou HH, Hu TQ, Liu Q, Ongodia D, Li ZB. (2012) Does trauma etiology affect the pattern of mandibular fracture? J Craniofac Surg 23: e494–497. 10.1097/SCS.0b013e3182646525 22976719

[27] Duan DH, Zhang Y (2008) Does the presence of mandibular third molars increase the risk of angle fracture and simultaneously decrease the risk of condylar fracture? Int J Oral Maxillofac Surg 37: 25–28. 17881190 10.1016/j.ijom.2007.07.010

[28] Thangavelu A, Yoganandha R, Vaidhyanathan A (2010) Impact of impacted mandibular third molars in mandibular angle and condylar fractures. Int J Oral Maxillofac Surg 39: 136–139. 10.1016/j.ijom.2009.12.005 20083388

[29] He D, Ellis E 3rd, Zhang Y (2008) Etiology of temporomandibular joint ankylosis secondary to condylar fractures: The role of concomitant mandibular fractures. J Oral Maxillofac Surg 66: 77–84. 18083419 10.1016/j.joms.2007.08.013

